# The Impact of Ayahuasca on Suicidality: Results From a Randomized Controlled Trial

**DOI:** 10.3389/fphar.2019.01325

**Published:** 2019-11-19

**Authors:** Richard J. Zeifman, Fernanda Palhano-Fontes, Jaime Hallak, Emerson Arcoverde, João Paulo Maia-Oliveira, Draulio B. Araujo

**Affiliations:** ^1^Centre for Psychedelic Research, Department of Brain Sciences, Faculty of Medicine, Imperial College London, London, United Kingdom; ^2^Brain Institute, Federal University of Rio Grande do Norte (UFRN), Natal, Brazil; ^3^Onofre Lopes University Hospital, UFRN, Natal, Brazil; ^4^Department of Neurosciences and Behaviour, University of Sa~o Paulo (USP), Ribeira~o Preto, Brazil

**Keywords:** suicidality, ayahuasca, psychedelics, randomized controlled trial, novel intervention

## Abstract

Suicide is a major public health problem. Given increasing suicide rates and limitations surrounding current interventions, there is an urgent need for innovative interventions for suicidality. Although ayahuasca has been shown to target mental health concerns associated with suicidality (i.e., depression and hopelessness), research has not yet explored the impact of ayahuasca on suicidality. Therefore, we conducted secondary analyses of a randomized placebo-controlled trial in which individuals with treatment-resistant depression were administered one dose of ayahuasca (*n* = 14) or placebo (*n* = 15). Suicidality was assessed by a trained psychiatrist at baseline, as well as 1 day, 2 days, and 7 days after the intervention. A fixed-effects linear mixed model, as well as between and within-groups Cohen's *d* effect sizes were used to examine changes in suicidality. Controlling for baseline suicidality, we found a significant effect for time (*p* < .05). The effect of the intervention (i.e., ayahuasca vs. placebo) trended toward significance (*p* = .088). At all time points, we found medium between-group effect sizes (i.e., ayahuasca vs. placebo; day 1 Cohen’s *d* = 0.58; day 2 d = 0.56; day 7 d = 0.67), as well as large within-group (ayahuasca; day 1 Cohen's *d* = 1.33; day 2 d = 1.42; day 7 d = 1.19) effect sizes, for decreases in suicidality. Conclusions: This research is the first to explore the impact of ayahuasca on suicidality. The findings suggest that ayahuasca may show potential as an intervention for suicidality. We highlight important limitations of the study, potential mechanisms, and future directions for research on ayahuasca as an intervention for suicidality.

**Clinical Trial Registration:** www.ClinicalTrials.gov, identifier NCT02914769.

## Introduction

Suicide is a public health issue of major concern: it is a leading cause of premature death, accounting for nearly one million deaths annually (World Health Organization, 2014). For every completed suicide, it is estimated that 20–30 suicide attempts occur (Wasserman, 2001). Furthermore, suicide rates have been increasing within the United States (Curtin et al., 2016).

Suicide occurs most commonly among individuals with major depressive disorder (MDD) (Cavanagh et al., 2003; World Health Organization, 2014) and individuals with comorbid MDD and borderline personality disorder (BPD) exhibit especially heightened levels of suicidality (Soloff et al., 2000; Galione and Zimmerman, 2010; Perugi et al., 2013; Zeng et al., 2015). Given the drastic consequences of suicide and suicide attempts, effective suicide interventions are of great importance.

A number of interventions are effective for treating suicidality (i.e., suicide attempts, suicide planning, and suicidal ideation; for a review, see Zalsman et al., 2016), including electroconvulsive therapy, psychotherapy (e.g., cognitive behavior therapy, dialectical behavior therapy), and pharmacological interventions (e.g., antidepressants, lithium, clozapine). However, there remain a number of important limitations surrounding current interventions for suicidality, including (a) non-immediate effects (e.g., weeks to months; Griffiths et al., 2014), (b) limited treatment availability (Bateman, 2012), (c) negative side-effects (e.g., increased suicidality with antidepressant use among adolescents; Vitiello and Ordóñez, 2016), (d) the need for ongoing administration (Kellner et al., 2005), and (e) high rates of non-responsiveness (Stone et al., 2009; Pompili et al., 2010). Individuals who do not respond to conventional interventions (i.e., individuals with treatment-resistant depression) show especially heightened levels of suicidality (Nelsen and Dunner, 1995; Malhi et al., 2005; Souery et al., 2007) and are, therefore, especially in need of novel interventions for suicidality.

### Novel Interventions for Suicidality

One novel intervention that has recently received attention for the treatment of suicidality is ketamine, a dissociative that acts as an antagonist of N-methylD-aspartate (NMDA). A recent meta-analysis (*k* = 10; *N* = 167; Wilkinson et al., 2018) of randomized controlled trials on the impact of a single dose of ketamine (vs. saline or midazolam) on suicidality found medium to large between-group effect sizes both 1 day [effect size (ES) = 0.85], 2 (ES = 0.85), and 7 days (ES = 0.61) after administration. Importantly, this meta-analysis suggests that the impact of ketamine on suicidality may begin to decrease within a week after administration. Moreover, there is no evidence that the antisuicidal effects of ketamine are long-lasting (Zalsman et al., 2016; Dadiomov and Lee, 2019) and there are significant concerns surrounding repeated administration of ketamine, including the potential for abuse and cognitive impairment (Schak et al., 2016; Strong and Kabbaj, 2018). Thus, there is a need for identifying alternative novel interventions for suicidality with less potential for abuse and a longer-lasting impact on suicidality.

One potentially promising novel intervention for suicidality, which has shown promise for a wide range of mental health concerns (for a review, see dos Santos et al., 2018) are psychedelics. Psychedelics are a class of pharmacological agents, including psilocybin and ayahuasca (a brew which contains N,N-dimethyltryptamine and beta-carboline alkaloids), that induce changes in affect, cognition, and perception, as well as non-ordinary states of consciousness at high doses (Griffiths et al., 2006; Hamill et al., 2019).

Cross-sectional and longitudinal research indicates that lifetime use of psychedelics is associated with lower levels of suicidality. For instance, among adult males (*N* = 190,000), lifetime psychedelic use was associated with lower levels of past-year suicide ideation, planning, and attempts (Hendricks et al., 2015). Furthermore, within a community-based cohort of marginalized women, lifetime psychedelic use was predictive of reduced risk of suicidality (Argento et al., 2017), as well as buffered the relationship between opioid use and suicidality (Argento et al., 2018). However, given that these studies were non-experimental, they leave open the question of whether these effects are due to factors associated with psychedelic use, such as personality, or whether *administration* of psychedelics leads to decreases in suicidality.

To date, only a single study has experimentally explored the impact of psychedelics on suicidality. Carhart-Harris and colleagues (2018) conducted an open-label trial in which individuals with treatment-resistant MDD received two doses of psilocybin with psychological support. Results indicated significant decreases in self-reported suicidality 1 and 2 weeks after the intervention. This study was limited by reliance upon self-reported suicidality and the open-label design. Accordingly, additional experimental research on the impact of psychedelics on suicidality, using clinician assessed suicidality and a placebo-controlled design, is necessary.

Additional support for the impact of psychedelics on suicidality comes from clinical research indicating that interventions that include administration of psilocybin (Carhart-Harris et al., 2016; Griffiths et al., 2016; Ross et al., 2016; Carhart-Harris et al., 2018) and ayahuasca (Santos et al., 2007; Thomas et al., 2013; Osório et al., 2015; Sanches et al., 2016; Palhano-Fontes et al., 2019; Uthaug et al., 2018) lead to acute and sustained reductions in mental health concerns associated with suicidality, such as depression and hopelessness (Brown et al., 2000; Nock et al., 2009). For instance, among individuals with treatment-resistant MDD, a recent randomized placebo-controlled trial showed large decreases in depressive symptoms 1, 2, and 7 days administration of ayahuasca (Palhano-Fontes et al., 2019). However, extant research suggests that suicidality can occur independent from depressive symptoms (Brent et al., 2005; Brent, 2010; Brent and Mann, 2010; Dutta et al., 2017; Batterham et al., 2019) and decreases in depressive symptoms are not always associated with decreases in suicidality (Christensen et al., 2013). For instance, compared with placebo, even first-line interventions for depression (i.e., selective serotonin reuptake inhibitors; SSRIs) lead to limited to no decreases in suicidality (intent to treat ES = –0.04–0.20; Näslund et al., 2018). Therefore, there is a need for research on the impact of ayahuasca directly on suicidality.

In sum, suicide is an increasingly problematic mental health concern and there are important limitations surrounding current interventions for suicidality. Lifetime psychedelic use is associated with lower levels of suicidality. Furthermore, ayahuasca and psilocybin have shown promise as interventions for a wide range of mental health issues associated with suicidality. However, research has not yet explored whether the administration of ayahuasca leads to reductions in suicidality. In order to fill this gap in the literature, we conducted secondary analyses of data from a randomized placebo-controlled trial, in which individuals with treatment-resistant MDD were administered a single dose of ayahuasca or placebo (see primary analysis: Palhano-Fontes et al., 2019). We hypothesized that ayahuasca would lead to decreases in suicidality that are sustained (i.e., from 1 to 7 days after the intervention). We also conducted exploratory analyses in order to determine whether changes in suicidality were associated with changes in non-suicide-related depressive symptoms.

## Methods

### Procedures

We conducted secondary analyses of a double-blind, parallel-arm, randomized placebo-controlled trial for individuals with treatment-resistant MDD (for primary outcomes, see Palhano-Fontes et al., 2019). Participants were recruited *via* referral from outpatient psychiatric units and advertisement. Interested participants received a full clinical assessment by a psychiatrist in order to determine eligibility. To be eligible to participate in the study, participants needed to be: between ages 18 and 60, meeting criteria for a unipolar MDD, which was assessed using the Portuguese version (Del-Ben et al., 2001) of the Structured Clinical Interview for DSM-IV (SCID-IV; First et al., 1997), and treatment-resistant (i.e., inadequate response to 2 or more antidepressant medications from different classes; Conway et al., 2017). Exclusion criteria for the study included: prior experience using psychedelics, current medical disease, pregnancy, imminent suicidal risk, or use of substances of abuse, current or previous neurological disorders, and personal or family history of schizophrenia, bipolar affective disorder, mania, or hypomania. Eligible participants were randomly assigned (1:1) to either the ayahuasca or placebo group, randomized in blocks of 10. Investigators and participants were blind to the treatment condition. Blindness was enhanced through the exclusion of individuals with past experience with ayahuasca, the use of an active placebo, as well as through randomly assigning participants to different assessors following the intervention.

Antidepressant medication was discontinued prior to intervention (average 2 weeks, dependant on the half-life of the antidepressant) and for 7 days post-intervention. Daily benzodiazepine use was permitted, excluding during the acute phases of the intervention. On the morning of the intervention, participants were reminded with information regarding potential experiences and strategies for dealing with difficult experiences during the ayahuasca inebriation. Participants were also instructed to focus on their bodies, thoughts, and emotions. The intervention occurred in a quiet and dimly lit environment with a bed and a recliner. Participants listened to a predefined music playlist throughout the intervention.

Within an individual setting, participants were administered a single 1 ml/kg dose of either ayahuasca (mean ± S.D.; 0.36 ± 0.01 mg/ml of N, N-DMT, 1.86 ± 0.11 mg/ml of harmine, 0.24 ± 0.03 mg/ml of harmaline, and 1.20 ± 0.05 mg/ml of tetrahydroharmine) or placebo (per 1 ml of water: 0.1 g of yeast, 0.02 g of zinc sulfate, and 0.02 g of citric acid). The placebo was designed to imitate the bitter/sour taste, brownish color, and gastrointestinal distress often present during the effects of ayahuasca. During the session, two investigators remained next door to provide support when needed. Sessions lasted approximately 8 h, after which participants were permitted to return home. Following the intervention, four participants opted to remain as inpatients throughout the 7-day period. Suicidality was assessed at (a) baseline, (b) 1 day, (c) 2 days, and (d) 7 days after the intervention. The study adhered to recommended clinical guidelines for safely conducting psychedelic administration (Johnson et al., 2008), it occurred at the Onofre Lopes University Hospital (HUOL), Natal-RN, Brazil, and was approved by the University Hospital Research ethics committee. For additional details regarding study procedures, including a CONSORT diagram of the trial profile, as well as the impact of ayahuasca on depressive symptoms, not including analyses of suicidality, see Palhano-Fontes and colleagues (2019).

## Measures

**Montgomery-Åsberg Depression Rating Scale—**The Montgomery-Åsberg Depression Rating Scale (MADRS; Montgomery and Asberg, 1979) is a 10-item, clinician-administered measure of depression severity. The measure includes one item (item 10; MADRS-suicidality item; MADRS-SI) that assesses current suicidality. MADRS-SI is rated on a scale from 0 to 6. The ratings are as follows: 0 (“Enjoys life or takes it as it comes.”), 2 (“Weary of life. Only fleeting suicidal thoughts.”), 4 (“Probably better off dead. Suicidal thoughts are common, and suicide is considered as a possible solution but without specific plans or intention.”), and 6 (“Explicit plans for suicide when there is an opportunity. Active preparations for suicide.”). Odd ratings (i.e., 1, 3, and 5) may be used but are not specifically defined. Past research has defined clinically significant suicidality as MADRS-SI ≥ 4 (Ballard et al., 2015). Assessment of suicidality using the MADRS-SI is common in suicidality research (e.g., Price et al., 2009; Larkin and Beautrais, 2011; Perroud et al., 2011; Price et al., 2014; Ballard et al., 2015) and is considered a valid approach for the assessment of suicidality (Desseilles et al., 2012). In line with past research (e.g., Price et al., 2014; Ballard et al., 2015), we used the sum of the remaining nine items of the MARDS to measure non-suicide-related depressive symptoms (MADRS-total_nonSI_).

### Statistical Analysis

We used a modified intention-to-treat analysis, in which all participants that received the intervention (i.e., ayahuasca or placebo) were included in analyses. We ran a fixed-effects linear mixed model, examining MARDS-SI scores 1 day, 2 days, and 7 days after the intervention, with baseline MADRS-SI scores as a covariate. We used an unstructured covariance structure and estimated missing data (1 and 2 days after the intervention two participants failed to attend assessments) with restricted maximum-likelihood estimation. We evaluated the main effects of time and intervention, as well as a time x intervention interaction. For MADRS-SI and MADRS-total_nonSI_ scores, we calculated between-group Cohen's *d* effect sizes by dividing estimated marginal means (1 day, 2 days, and 7 days after the intervention) for each group by pooled standard deviations. We also calculated within-group Cohen's *d* effect sizes by dividing change scores (time point—baseline) at each time point (1 day, 2 days, and 7 days after the intervention) by the standard deviation of the change score. For within-group effect sizes, missing values were not imputed. For the relationship between changes in MADRS-SI and MADRS-total_nonSI_ scores 7 days after the intervention, within both the ayahuasca and placebo groups, we calculated Pearson correlation coefficients. We set the alpha level indicating significance at *p* < 0.05, two-tailed. All analyses were conducted using IBM SPSS Statistics (Version 25).

## Results

### Demographics

Participant mean age was 42.04 (*SD* = 11.66). The majority of participants were female (72%), Caucasian (59%), unemployed (52%), had a lifetime suicide attempt (55%), and a comorbid personality disorder (76%). Nine individuals (31%) had a diagnosis of BPD. At baseline, participant mean MADRS-SI score was 2.35 (*SD* = 1.91). For additional details regarding participants' characteristics by condition, see **Table 1**. For individual participant details related to the presence of a personality disorder and MADRS-SI at each time point, see **Table 2**.

**Table 1:** Sample characteristics and treatment history by study condition.

Variable	Ayahuasca group (*n* = 14)	Placebo group (*n* = 15)
Age *M* (*SD*)	39.71 (11.26)	44.2 (11.98)
Sex-Female	11	5
Ethnicity		
Black	1	0
Pardo	4	7
Caucasian	9	8
Education		
Incomplete Primary Education	5	4
Completed Primary Education	1	2
Completed Secondary Education	4	7
Completed Undergraduate Education	1	0
Completed Postgraduate Education	3	2
Employment status		
Employed-working	3	3
Employed-leave of absence	2	4
Unemployed	7	8
Student	2	0
Presence of personality disorder	10	12
Borderline personality disorder	5	4
Dependant personality disorder	1	0
Histrionic personality disorder	4	7
Narcissistic personality disorder	1	0
Schizoid personality disorder	0	1
Cluster B (undefined)	1	0
Depression duration (years)		
Baseline MADRS-SI *M* (*SD*)	3.36 (1.65)	1.40 (1.68)
Lifetime suicide attempt	10	6
Number of failed antidepressant medications *M* (*SD*)	3.93 (1.44)	3.80 (1.90)
History of psychotherapy	11	12
History of electroconvulsive therapy	1	1
Inpatient during the intervention	2	2

Cluster B personality disorders include antisocial personality disorder, borderline personality disorder, histrionic personality disorder, and narcissistic personality disorder.

**Table 2:** Participant clinical characteristics.

Condition	Personality disorder	Suicidality (MARDS-SI)			
		Baseline	1 day post	2 days post	7 days post
A1	Histrionic	4	4	4	4
A2	Histrionic	2	0	0	0
A3	Borderline	4	3	*	2
A4	Histrionic + Dependent	3	0	0	0
A5	Cluster B (undefined)	0	0	0	0
A6	None	3	0	0	0
A7	Borderline + Narcissistic	5	1	1	1
A8	Histrionic	4	2	0	2
A9	None	5	1	0	0
A10	None	0	0	0	0
A11	Borderline	5	0	0	0
A12	None	4	3	3	0
A13	Borderline	4	2	0	0
A14	Borderline	4	0	1	5
P1	Schizoid	2	0	0	0
P2	Histrionic	0	0	0	0
P3	Borderline	5	2	2	2
P4	None	0	4	0	2
P5	Borderline	3	*	3	3
P6	Histrionic	1	0	0	0
P7	None	0	0	0	0
P8	Borderline	3	1	1	1
P9	None	2	1	1	1
P10	Histrionic	0	0	0	0
P11	Histrionic	0	0	0	0
P12	Histrionic	0	0	0	2
P13	Histrionic	4	*	*	4
P14	Borderline	1	2	1	5
P15	Histrionic	0	3	0	0

MADRS-SI, Montgomery-Asberg Depression Rating Scale-Suicidality Item; A, ayahuasca; P, placebo; *, Failed to attend assessment.

### Clinical Response

**Figure 1:**
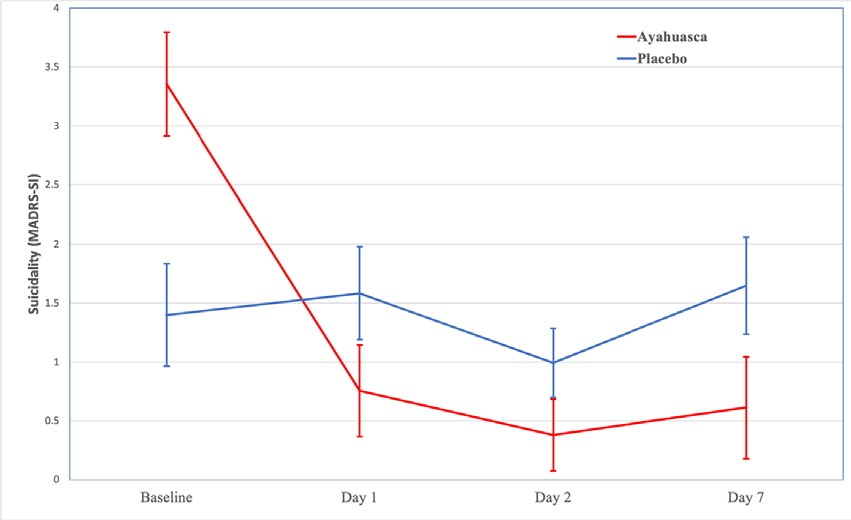
Changes in suicidality (MADRS-SI) over time. Baseline values are means (± standard error of the mean) for MADRS-SI. Day 1, Day 2, and Day 7 values are estimated marginal means (±standard error of the mean) for MADRS-SI.

For changes in suicidality (MADRS-SI) by group, see **Figure 1**. Results of the linear mixed model showed a significant effect for time, *F*(2,25.72 = 3.38; *p* < .05) and a trend toward significance for the intervention (i.e., ayahuasca vs. placebo), *F*(1,27.44 = 3.13; *p* = .088). The interaction between time and intervention was not significant, *F*(3,25.72 = .395; *p* = .678).

We found medium between-group (ayahuasca vs. placebo) effect sizes for decreases in MADRS-SI 1 day (Cohen’s *d* = 0.58; 95% CI –1.32–0.17), 2 days (Cohen's *d* = 0.56; 95% CI –1.30–0.18), and 7 days (Cohen's *d* = 0.67; 95% CI –1.42–0.08) after the intervention (see **Table 3**). Within the ayahuasca group, we found large within-group effect sizes for decreases in MADRS-SI 1 day (Cohen's *d =* 1.33; 95% CI 1.25–3.18; *n* = 14), 2 days (Cohen's *d =* 1.42; 95% CI 1.50–3.74; *n* = 13), and 7 days (Cohen's *d =* 1.19; 95% CI 1.21–3.50; *n* = 14) after the intervention. Within the placebo group, we found small within-group effect sizes for decreases in MADRS-SI 1 day (Cohen's *d =* 0.00; 95% CI –1.09–1.63; *n* = 13) and 7 days (Cohen's *d =* 0.04; 95% CI –0.90–1.04; *n* = 15) after the intervention, as well as a medium effect size 2 days (Cohen's *d =* 0.64; 95% CI 0.06–1.23; *n* = 14) after the intervention (see **Table 4**). Overall, these effect sizes suggest that ayahuasca leads to decreases in suicidality that are sustained from 1 to 7 days after administration. For between and within-group effect sizes for changes in non-suicide-related depressive symptoms (MADRS-total_nonSI_), see **Tables 3** and **4**, respectively.

**Table 3:** Between-group effect sizes (Cohen's d) for suicidality (MADRS-SI) and non-suicide-related depressive symptoms (MADRS-total_nonSI_)

	Day 1	Day 2	Day 7
MADRS-SI			
Cohen's *d*	0.58	0.56	0.67
95% CI	–1.32–0.17	–1.30–0.18	–1.42–0.08
**MADRS-total**_nonSI_			
Cohen's *d*	0.59	0.65	1.55
95% CI	–1.33–0.16	–1.40–0.10	–2.38–0.72

**Table 4:** Mean scores and within-group effect sizes (Cohen’s d) for suicidality (MADRS-SI) and non-suicide-related depressive symptoms (MADRS-total_nonSI_).

	Baseline		Day 1		**Day 2**		**Day 7**	
	Aya	Pla	Aya	Pla	Aya	Pla	Aya	Pla
***n***	14	15	14	13	13	14	14	15
**MADRS-SI**								
Mean	3.36	1.40	1.14	1.00	0.69	0.57	1.00	1.33
*SD*	1.65	1.68	1.41	1.35	1.32	0.94	1.66	1.63
*Cohen's d*	–	–	1.33	0.00	1.42	0.64	1.19	0.04
95% CI	–	–	1.25–3.18	–1.09–1.63	1.50–3.74	0.06–1.23	1.21–3.50	–0.90–1.04
**MADRS-total**_nonSI_								
Mean	32.79	28.73	15.57	15.00	13.54	13.86	14.64	22.20
*SD*	4.93	5.08	12.68	11.16	10.07	10.04	8.64	9.87
*Cohen's d*	–	–	1.46	1.61	2.17	1.57	2.20	0.91
95% CI	–	–	10.42–24.02	8.06–17.78	14.03–24.89	8.94–19.35	13.38–22.92	2.55–0.51

Aya, ayahuasca; Pla, placebo.

### Association Between Changes in Suicidality and Depressive Symptoms

Seven days after the intervention, within the ayahuasca group, the association between changes in changes in suicidality (MADRS-SI) and changes in non-suicide-related depressive symptoms (MADRS-total_nonSI_) approached significance, *r* = .53, *p* = .053. Within the placebo group, the association between changes in suicidality (MADRS-SI) and changes in non-suicide-related depressive symptoms (MADRS-total_nonSI_) was not significant, *r* = .16, *p* = .579.

## Discussion

Given the limitations surrounding current interventions, there is an urgent need for innovative interventions for suicidality (Research Prioritization Task Force, 2014). To date, experimental research had not yet directly explored the impact of ayahuasca on suicidality. Therefore, this study aimed to fill this gap by being the first to explore the impact of ayahuasca on suicidality.

We hypothesized that ayahuasca would lead to decreases in suicidality that are sustained (i.e., from 1 to 7 days after the intervention). Our results are mixed. Although across groups there was a significant decrease in suicidality over time, the effect for the treatment group (i.e., ayahuasca vs. placebo) trended toward but did not reach significance. There are a number of potential explanations for these results. First, compared with placebo, ayahuasca may not lead to significant decreases in suicidality. Given that research has not yet explored the direct impact of ayahuasca on suicidality, this is a plausible explanation. Alternatively, given that large effects may not be detected by statistical tests, especially within small sample sizes (Greenland et al., 2016), our study was likely underpowered to detect the impact of ayahuasca (compared with placebo) on suicidality. Therefore, consideration of effect sizes is essential for the interpretation of our findings. In support of this possibility, we found medium between-group effect sizes for decreases in suicidality at all time points. Furthermore, within the ayahuasca group, we found large effect sizes for decreases in suicidality at all time points. These findings are in line with past research on the impact of psilocybin on suicidality (Carhart-Harris et al., 2018), as well as cross-sectional (Hendricks et al., 2015) and longitudinal (Argento et al., 2017) research indicating that lifetime use of psychedelics is associated with reduced levels of suicidality and decreased risk of becoming suicidal. Furthermore, these results are in line with past research indicating that the administration of ayahuasca is associated with improvement in mental health concerns associated with suicidality (e.g., depression and hopelessness; Santos et al., 2007; Palhano-Fontes et al., 2019). Interestingly, 7 days after the intervention, between and within-group effect sizes for decreases in non-suicide-related depressive symptoms were larger than those found for suicidality, which may suggest that ayahuasca has a greater impact on non-suicide-related depressive symptoms than suicidality. Alternatively, these results may be due to a floor effect as a result of low levels of baseline suicidality. Nonetheless, overall, these results suggest that the therapeutic benefits of ayahuasca may extend to suicidality and that investigation of the impact on ayahuasca on suicidality using a larger sample is warranted.

These findings are also important as they indicate that ayahuasca may have a fast-acting impact on suicidality (i.e., as soon as 1 day after the intervention). Given that the time between the emergence of suicidality and suicide can be very short (Deisenhammer et al., 2009), there is a need for fast-acting interventions for suicidality. Currently, recommended interventions for suicidality are limited by the duration of time they take to be effective. For instance, individuals with MDD that are treated with antidepressants remain at high risk of suicide for at least 10–14 days after treatment begins (Jick et al., 2004; Simon et al., 2006). Furthermore, compared with nonsuicidal individuals, individuals with moderate to high levels of suicidality show slower responses to antidepressants (Baldessarini et al., 2006). Similarly, among individuals receiving ECT three times a week, suicidality often persists for 1–2 weeks after intervention (Kellner et al., 2005). Similar to the fast-acting effects of ketamine on suicidality (Bartoli et al., 2017), ayahuasca may also show promise as a fast-acting intervention for suicidality.

We found similar medium between-group and large within-group effect sizes for decreases in suicidality at all time points, with the largest between-group effect size 7 days after the intervention. These results suggest that the impact of ayahuasca on suicidality may last beyond the acute and post-acute effects of ayahuasca. These findings are in line with past research indicating that ayahuasca (e.g., Sampedro et al., 2017; Sanches et al., 2016; Palhano-Fontes et al., 2019); and psilocybin (e.g., Griffiths et al., 2016; Ross et al., 2016; Carhart-Harris et al., 2018) lead to mental health improvements that last beyond their acute effects. These results are especially important in light of the need for ongoing treatment in interventions for suicidality. For instance, ECT (Tanney, 1986; Prudic and Sackeim, 1999; Kellner et al., 2005) and traditional interventions for suicidality require ongoing administration in order to maintain their antisuicidal effects (Valuck et al., 2009). Similarly, research suggests that ketamine also requires repeated administration in order to maintain its efficacy (Zalsman et al., 2016; Dadiomov and Lee, 2019), which is problematic given the potential for cognitive impairment and abuse with repeated administration of ketamine (Schak et al., 2016; Strong and Kabbaj, 2018). Importantly, ayahuasca is associated with a low abuse and dependence potential (Hamill et al., 2019). Therefore, these findings suggest that ayahuasca may show promise as an intervention for suicidality that does not require repeated administration. Additional research with longer-term follow-up will be necessary to determine the long-term impact of ayahuasca on suicidality.

Interestingly, within the ayahuasca group, the relationship between changes in suicidality and changes in non-suicide-related depressive symptoms approached significance, with a large effect size (i.e., *r* = .53). These findings suggest that the impact of ayahuasca on suicidality may, in part, be due to its impact on non-suicide-related depressive symptoms or mechanisms overlapping both non-suicide-related depressive symptoms and suicidality. Research suggests that suicide functions as a means of escaping intense emotional distress (Baumeister, 1990; Shneidman, 1998). Extant research indicates that psychedelics in general, and ayahuasca in particular, leads to decreases in emotional distress (for a review, see dos Santos et al., 2018). Similarly, a recent study found that the administration of ayahuasca led to decreases in emotion dysregulation, within a community sample and among individuals with BPD traits (Domínguez-Clavé et al., 2019). Similarly, among males in a community sample, lifetime use of psychedelics was associated with lower levels of emotion dysregulation (Thiessen et al., 2018). One particular means through which ayahuasca may decrease emotion dysregulation is *via* increased mindfulness-related capacities (e.g., acceptance and decentering), which have been shown to increase after administration of ayahuasca (Thomas et al., 2013; Soler et al., 2016; Sampedro et al., 2017; Domínguez-Clavé et al., 2019; Soler et al., 2018; Uthaug et al., 2018). Neurobiological research similarly suggests that ayahuasca may impact suicidality *via* decreases in emotion dysregulation. For instance, among individuals with MDD, a single dose of ayahuasca led to increased blood flow in regions of the brain associated with emotion regulation (e.g., left nucleus accumbens, right insula and left subgenual area; Sanches et al., 2016). Furthermore, research has found that administering psychedelics to rats promotes neuroplasticity (Ly et al., 2018), and markers of neuroplasticity (Nichols and Sanders-Bush, 2002), within the prefrontal cortex, a region of the brain implicated in emotion dysregulation (Vollenweider and Kometer, 2010) and suicidality (Ding et al., 2015). Therefore, the impact of ayahuasca on suicidality may be accounted for by its impact on psychological and neurobiological mechanisms associated with emotion dysregulation. Additional research is necessary in order to understand the mechanisms that account for the impact of ayahuasca on suicidality.

### Ayahuasca and Borderline Personality Disorder

One psychiatric disorder that ayahuasca may show promise as an intervention for is BPD, a severe psychiatric disorder associated with especially high rates of suicide (i.e., 3%–10%; Links 2009) and suicide attempts (i.e., 60%–78%; Links 2009). Importantly, there is limited evidence for the efficacy of treating BPD with pharmacological agents and a pressing need for innovative pharmacological interventions for BPD (Chanen, 2015; Starcevic and Janca, 2018). Interestingly, among individuals with BPD traits, a recent study found that the administration of ayahuasca led to decreases in components of emotion dysregulation (Domínguez-Clavé et al., 2019), which is considered the core dysfunction in BPD (Linehan, 1993; Carpenter and Trull, 2013). However, they did not include a sample of individuals that met diagnostic criteria for BPD and the impact of ayahuasca on suicidality was not assessed. The present study was the first clinical trial with psychedelics to report including individuals with BPD. It is noteworthy that no serious adverse events occurred among individuals with BPD. Furthermore, while all five individuals with BPD that received ayahuasca showed clinically significant suicidality at baseline (i.e., MADRS-SI ≥ 4), none (i.e., 0%) reported clinically significant suicidality 1 and 2 days after administration and only 1 (i.e., 20%) reported clinically significant suicidality 7 days after administration (see **Table 2**). Given the limited number of individuals with BPD included in the sample, these results must be interpreted with caution. However, given the pressing need for innovative pharmacological interventions for BPD, the wide-ranging mental health concerns for which psychedelics have shown promise, and our results related to the impact of ayahuasca on suicidality, additional research exploring the safety, tolerability, and clinical utility of ayahuasca as an intervention for BPD may be warranted.

### Strengths, Limitations, and Future Direction

This study includes a number of strengths and limitations that are important to consider. The study utilized a double-blind randomized placebo-controlled design, a gold-standard for suicide research (Zalsman et al., 2016). Furthermore, in order to increase blinding, the study only included individuals without past experience with psychedelics and used an active placebo designed to imitate characteristics of ayahuasca. Additionally, the sample used in the present study showed severe psychiatric comorbidity, with the majority of the sample (76%) meeting criteria for a comorbid DSM-IV axis II disorder. Furthermore, all individuals had treatment-resistant MDD and some had failed to respond to as many as 10 interventions. The impact of ayahuasca on suicidality among individuals with such high rates of non-responsiveness to conventional interventions is especially promising.

The primary limitation surrounding the present study is that, despite the use of randomization, suicidality among those in the placebo group was low. Therefore, although, we controlled for baseline differences in suicidality, the possibility that our results may be influenced by the placebo effect or regression to the mean, cannot be ruled out. Nonetheless, given the large within-group effects we found, we suggest that additional placebo-controlled research on the impact of ayahuasca on suicidality will be important. Relatedly, our study is limited by the use of a small sample size, which limits the extent to which inferential statistics are able to identify significant changes. Furthermore, due to the small sample size, potentially important covariates (e.g., use of benzodiazepines) were not included in our analyses. Future research would benefit from studies with larger samples that are better powered to detect the impact of ayahuasca on suicidality. Second, due to safety concerns, the study did not include individuals with imminent suicide risk. Accordingly, it is difficult to determine from the present study whether ayahuasca would lead to reductions in suicidality among individuals with higher levels of suicidality. Past research on pharmacological interventions for suicidality has employed similar exclusion criteria (e.g., Price et al., 2009) and the suicidality exclusion criteria employed in this study were less strict than past studies on pharmacological interventions for suicidality (e.g., MADRS-SI > 4; Ballard et al., 2015). Future research would benefit from exploring the impact of ayahuasca on individuals with higher levels of suicidality. Third, we did not conduct a qualitative analysis of participants descriptions of *why* ayahuasca impacted suicidality. Future research would benefit from using a mixed-methods approach, as well as analysis of potential mediators of the impact of ayahuasca on suicidality. Finally, similar to all research on psychedelics, due to the psychological experience induced by ayahuasca, ensuring that participants are blind to treatment condition is difficult (Barnby and Mehta, 2018). In order to improve blinding procedures in psychedelic research, future research should continue to develop increasingly convincing placebos.

This study was the first to explore the impact of ayahuasca on suicidality and our findings suggest that ayahuasca may show promise as a fast-acting and innovative intervention for suicidality. Given important limitations of our study (e.g., small sample size, low levels of baseline suicidality), additional research will be necessary in order to determine the long-term impact of ayahuasca on suicidality, as well as the safety, tolerability, and clinical outcomes associated with administration of ayahuasca among highly suicidal individuals.

## Data Availability Statement

The datasets generated for this study are available on request to the corresponding author.

## Ethics Statement

The studies involving human participants were reviewed and approved by Onofre Lopes University Hospital (HUOL), Natal-RN, Brazil, University Hospital Research Ethics Committee. The patients/participants provided their written informed consent to participate in this study.

## Author Contributions

FP-F, JH, EN, JM-O, and DA contributed to study design and conception. FP-F and DA coordinated data acquisition. RZ, FP-F, and DA analyzed data and interpreted the results. RZ was responsible for the first draft of the manuscript. All authors read, critically revised, and approved the manuscript.

## Funding

Funded by the Brazilian National Council for Scientific and Technological Development (CNPq, grants #466760/2014 & #479466/2013), and by the CAPES Foundation (grants #1677/2012 & #1577/2013).

## Conflict of Interest

The authors declare that the research was conducted in the absence of any commercial or financial relationships that could be construed as a potential conflict of interest.
